# Ante-Natal and Post-Natal Influences on Neonatal Immunity, Growth and Puberty of Calves—A Review

**DOI:** 10.3390/ani11051212

**Published:** 2021-04-22

**Authors:** Claudia L. Cardoso, Ailbhe King, Aspinas Chapwanya, Giulia Esposito

**Affiliations:** 1 Ruminant Health and Production, Department of Production Animal Studies, Faculty of Veterinary Science, University of Pretoria, Onderstepoort, 0110 Pretoria, South Africa; dracardoso@gmail.com; 2 Department of Clinical Sciences, Ross University School of Veterinary Medicine, Farm Road, 42123 Basseterre, Saint Kitts and Nevis; aking@rossvet.edu.kn (A.K.); achapwanya@rossvet.edu.kn (A.C.); 3 RUM&N Sas, Via Sant’Ambrogio, 42123 Reggio Emilia (RE), Italy; 4 Department of Animal Sciences, Stellenbosch University, 7600 Stellenbosch, South Africa

**Keywords:** calf immunology, colostrum, dam-foetus interaction, growth, gut health, puberty

## Abstract

**Simple Summary:**

The objective of this review is to give the reader an overview of interactions between immunity, growth and puberty in calves and highlight how these influence future performances. The risk of morbidity and mortality is high during the first four weeks of age. Adaption to extra-uterine life starts early during embryonic development and is underpinned by optimal maternal nutrition. It is known that colostrum is paramount to neonate nutrition and passive immunity. Good colostrum management allows the calf to develop coping mechanisms to efficiently utilize feed resources for optimal growth. A deeper understanding of these interactions paves the way for developing strategies to improve immune responses to environmental pathogens, optimal growth and timely attainment of puberty in calves. The literature reviewed here shows that there are opportunities to enhance future performance of cattle paying attention to the interaction of nutrition and immunity at early developmental stages. This then guarantees efficient neonate nutrition and profitable cattle production.

**Abstract:**

Calf immunity, growth and puberty are important factors affecting heifer productivity. The first four weeks of age are critical for reducing calf morbidity and mortality. It is well documented that colostrum is paramount to neonatal nutrition and passive immunity, however, adaptation to extra-uterine life starts early during embryonic development. Therefore, successful calf rearing strategies are underpinned by adequate maternal nutrition during gestation, and good colostrum management. A deeper understanding of these interactions paves the way for developing strategies to improve immune responses to environmental pathogens, optimal growth and timely attainment of puberty in calves. The literature reviewed here shows that there are opportunities to enhance the future performance of cattle paying attention to the interaction of nutrition and immunity at each developmental stage. Therefore, the objective of this review is to give the reader an overview of interactions between immunity, growth and puberty in dairy calves and highlight how these influence future performances.

## 1. Introduction

Development from conception to puberty progresses through well-regulated stages at molecular, cellular and animal level. For the neonate, passive transfer of immunity is a key primary defence mechanism against infections. Calf immunity, growth and puberty are critical factors influencing productivity of heifers. Some studies focused on these factors individually, rather than their integrations. Recently, considering the interactions between these vital physiological processes has become important to optimize calf survival. During gestation, the placenta supports foetal development and prepares it for extra-uterine survival [1]. After birth, colostrum transfers immune molecules [2,3] which, in case of failure, causes increased susceptibility to infections that lowers survival [4,5]. Colostrum also provides many growth factors such as insulin-like growth factor I (IGF-I) [6], a key factor regulating growth and immune response in calves [7].

Acquiring growth factors improves calf immunity because these factors modulate development and differentiation in-utero [8,9] as well as after birth. Colostrum stimulates gastrointestinal development and function through the absorption of growth factors and other immune components [10]. Therefore, good colostrum management is important to promote growth and daily weight gain [11,12] as well as facilitate early attainment of puberty.

A better understanding of the interrelationship of the factors involved in immune function, growth and reproduction is needed. The objective of this review is to discuss calf physiology and the interactions that enhance calf survival and to offer an insight into strategies adopted at these life stages to optimise production.

## 2. Dam-Foetus Interaction and Ante-Natal Development

Dam-foetus interaction through the placenta guarantees the survival of the foetus. During pregnancy, maternal metabolic adjustment to promote foetal development and growth occurs through two pathways: one represented by nutritional partitioning, and the other by increased placental development leading to improved nutrient transfer and hormonal production. Nutrient availability is also supported by appetite changes of the dam in order to support the growing foetus. It is thought that there are no negative effects of dietary protein content on any of the reproductive measures such as days to conception, calving interval [13]. However, diets containing balanced metabolizable energy and protein that satisfy both maintenance and growth requirements of the dam and conceptus growth are fundamental for optimal foetal development. Additionally, the placenta not only provides nutrients to the foetus, but it also regulates maternal cortisol levels that has a negative impact on foetal growth. Furthermore, these actions signal the placenta to alter foetal phenotype according to the current micro-uterine environment as an adaptive response (prenatal programming) [14]. In animal and biomedical science, there is growing evidence that foetal programming can alter postnatal development, growth, and disease susceptibility of the offspring [14]. Key times during which foetal programming can be altered are thought to be during placentation, immune system development and muscle and fat development.

### 2.1. Placentation

While most foetal growth occurs during late gestation [15], inadequate nutrition during early gestation can have profound effects on placental development, vascularization, and embryo organogenesis [16]. The placenta is a transient unique organ of pregnancy that provides an interface for metabolic exchange between the dam and the foetus [17]. It is a metabolically and immunologically active tissue that develops after maternal recognition of pregnancy in mammals [18], and it is fully developed by 40 days in cattle [17]. Placental surface growth, vascularisation and secretion of growth factors are maintained throughout gestation to meet increasing foetal demands. Thus, any nutrient restrictions during this phase can dramatically impair placentation and embryo development [19]. In fact, it was observed that nutrient restriction during early gestation, followed by adequate feeding during day 125 to 250 impaired placental angiogenesis and resulted in underdevelopment of placentomes [20]. Uteroplacental blood flow is central in programming foetal growth as it affects the transport of nutrients through the placenta. In fact, in large animal models it has been demonstrated that nutrients transport increases throughout gestation primarily because of increased uteroplacental blood flow rather than increased nutrient extraction from each unit of blood [21].

### 2.2. Immune System Development

The immune system of mammalian embryos starts developing early in gestation and continues to mature after birth [22,23]. Although the ruminant immune system forms during foetal development, the passive transfer of immunity depends mainly on quality and quantity of colostrum available and on the absorption capacity of the new-born [24,25,26,27]. However, research has demonstrated that the ability of the offspring to acquire immunoglobulins G (IgG) seems to be affected by the nutrition of the dam during late gestation [28]. In fact, a decrease in protein intake during the last trimester of gestation results in impaired serum IgG concentration in the calf. Furthermore, calves born to cows fed balanced diet, but fed colostrum from dams fed restricted diets have less serum IgG concentrations at 24 h of life than the calves receiving colostrum from well-nourished cows [28]. Nutrient-restricted cattle have decreased colostrum tri-iodothyronine concentration which plays an important role on IgG absorption at calf intestinal level [29,30].

### 2.3. Growth and Attainment of Puberty

Insulin growth factors (IGFs) influence maternal metabolism, thereby controlling nutrient availability for foetal growth. IGFs also regulate placental morphogenesis, substrate transport and hormone secretion. Increased IGF-I, secondary to maternal GH treatment in early pregnancy, increases maternal nutrient concentrations and sometimes increases foetal weight at the expense of maternal tissue mass [31]. The effects of the IGFs on foetal growth must occur indirectly either affecting maternal metabolism and nutrient partitioning, and/or placental development and function, since IGFs and GH do not cross the placenta in significant quantities [32].

The major factors controlling the onset of puberty are body weight and growth rather than age [33]. Some studies have demonstrated the importance of dam nutrition on growth, attainment of puberty and reproductive performance of the offspring. For instance, although heifers born to dams supplemented with protein during the last third of pregnancy had similar birthweight to calves born to unsupplemented dams, when mature these animals had higher pregnancy rates [34]. Restricted nutrition in beef cattle decrease birth weights and reduces postnatal growth [35]. Although the effect of global nutrients restrictions and /or excess has been extensively investigated, individual nutrients hinder offspring wellbeing if deficient. For example, methionine supplementation in Holstein cows changes the transcriptome of flushed embryos, including genes involved in embryonic development and immune responses [36]. Recent, research focusing on the effect of maternal nutrition on the neuroendocrine system function concluded that maternal nutrition modulates the hypothalamic pathways controlling GnRH release, and therefore affects the programming of puberty in the female offspring [37,38]. In cattle, little is known about the effects of dam nutrition on the neonate neuroendocrine system. Any nutritional and metabolic change occurring from foetal life to puberty, can impact the hypothalamic pathways controlling GnRH secretion and pubertal maturation [39].

In fact, maternal nutrition meeting the animal requirements during gestation, together with high body weight gain rates during early postnatal development, results in increased circulating levels of leptin, insulin, and IGF1; reduced neuropeptide Y (NPY) mRNA abundance and NPY (inhibitory) inputs to GnRH neurons. These endocrine and neuroendocrine changes, some of which will be discussed in Section 3.3.6, contribute to promoting early puberty.

### 2.4. Muscle and Fat Development

Skeletal muscle development is initiated in the embryonic stage, during the first trimester of pregnancy, when primary myogenesis occurs with formation of primary myofibers [40]. During this phase, maternal nutrition has negligible effects on foetal skeletal muscle development. In cattle the majority of muscle fibres form between the 2nd and 7th/8th months of gestation, period that corresponds to the secondary myogenesis [41]. This period is crucial because there will be no net increase in the number of muscle fibres after birth [42,43].

Post-natal muscle growth occurs mainly due to an increase in muscle fibre size rather than new muscle fibre formation [43,44]. Adipogenesis is initiated around mid-gestation [45], overlapping with the period of secondary myogenesis. The amount of intramuscular fat is determined by the number and size of intramuscular adipocytes within foetal skeletal muscle. Skeletal muscle and fat are less of a priority in terms of nutrient partitioning during foetal development when compared with organs such as the brain, heart, and liver. As a result, skeletal muscle development is particularly vulnerable to nutrient availability [46].

It has been reported that calves from nutrient restricted beef cows have decreased average daily gain and fatter carcasses at 30 months of age [47,48]. However, in beef calves from dams fed nutrient restricted diets during early pregnancy, but that were realigned during late gestation; muscle fibre size and muscle progenitor cell numbers could be rescued [49]. Furthermore, it has been reported that improving the dams’ diet by either increasing forage quality or exceeding their energy requirements during mid to late gestation, resulted in calves that were leaner and had better yielding carcasses compared to offspring from dams fed a negative energy diet [50,51].

## 3. Post-Natal Development

### 3.1. Immune Response

Ruminants possess a well-developed immune system at birth but are only capable of displaying a weak response against environmental pathogens. The ruminant foetus is protected by a syndesmochorial placenta that limits pathogen exposure in utero and does not permit placental immunoglobulin transfer [6] thus, rendering the offspring agammaglobulinemic [23,25]. Therefore, the innate immune system of the neonate is largely responsible for the defence of the organism at birth. However, during adulthood, these animals depend on both, innate and acquired immune response for survival, disease protection, and production efficiency [23,25]. Hence, because ruminants are born with no serum Igs, they are dependent on colostrum intake in the first few hours of life.

### 3.2. Colostrum Composition and Its Role on Passive Immunity Transfer and Neonatal Development

Colostrum contains important biomolecules. These include carbohydrates (lactose and oligosaccharides); proteins (casein, immunoglobulins-IgG more abundantly, α and β lactoglobulin, albumin, lactoferrin); growth factors (mainly insulin-like growth factor I and II-IGF-I, IGF-II-); enzymes (proteinases, lipases and esterases); enzyme inhibitors (macroglobulin, antithrombin, α-trypsin inhibitors); nucleotides and nucleosides (non-protein-nitrogen); cytokines (peptides or glycoproteins such as interleukins, interferons and growth hormone-GH-); lipid, minerals and vitamins [3]. In order to confer immunity, high quality colostrum must contain at least 50 g/L total IgG with adequate fat (20–30%), protein (15%), vitamins and minerals levels [52]. Additionally, it must have low bacterial contamination (<100.000 cfu/mL total bacteria count and <10.000 cfu/mL coliform count) and be free from colostrum transmissible pathogens (i.e., *Mycobacterium* spp., *Mycoplasma* spp. and *Salmonella* spp.) [53]. The key function of colostrum is to transfer passive immunity [6,24], including cells of immune origin such as lymphocytes [54]. Igs from the dam start to become available in receptors present in mammary alveolar tissue about fifteen days before parturition [55] and IgG represents 70 to 80% of the total immunoglobulins present in colostrum [3]. These molecules accumulate in the mammary gland by crossing the alveolar epithelium and reaching concentrations 5–10 times higher than in maternal serum [6,52]. In bovine colostrum about 75 to 90% of the IgGs (40 to 200 mg/mL) is represented by IgG1 [3,6,56,57] which represents the principal colostrum antibody transferred via the enteric cell to the neonate serum [3,6]. Various studies indicate as benchmark for successful passive transfer of immunity IgG concentrations of ≥10 g/L, based on the fact that below this level, mortality rates in calves increase due to failure of passive transfer [56,57,58,59]. However, as reported in the recent study conducted by Lombard et al. [59], the previously accepted IgG serum level of 10 g/L, is a way too simplistic benchmark to indicate transfer of passive immunity in calves. This because, although, as stated by the authors, mortality in calves has been reduced (most probably more due to better calf’ management than to better colostrum management) over the years, this is not always the case for morbidity. Therefore, from the analyses of data from 2545 heifer calves from 104 different operations, the authors proposed four serum IgG categories indicating the level of passive transfer of immunity as excellent, good, fair, and poor with serum IgG levels of ≥25.0, 18.0–24.9, 10.0–17.9, and <10 g/L, respectively. [59]. Hence, hindering of the absorption process leading to reduced serum IgG levels may predispose the new-born to debilitating digestive and/or respiratory infections that may lead to septicaemia and early mortality [58,60].

Other important components of colostrum are the peptides, which stimulate cell differentiation and growth. Amongst these components, the most revised in the literature are insulin-like growth factor I (IGF-I), insulin-like growth factor II (IGF-II) as well as the glycoprotein (cytokine group) and growth hormone (GH) [10]. If consumed within 6 h of calving, colostrum IGF-I and GH are 870 ng/mL^−1^ and 0.17 ng/mL, respectively, and decline over a period of 4 days to 16% of the initial level [61]. These peptides build up in the mammary gland as well shortly before parturition. They bind to IGF (I and II) and GH receptors present in the mammary gland. IGF receptors mediate IGFs transport to alveolar spaces from which they will be passed to the new-born via colostrum [62]. Igs, cytokines, immune cells (mainly lymphocytes) and growth factors are the main components important for calf survival over the first days of life providing maturational, immunomodulatory, anti-inflammatory and anti-microbial effects [6,60].

### 3.3. Intestinal Absorption of Colostrum Components

Absorption of colostrum in the small intestine at the apical tubular surface occurs for a limited time period after birth. Uptake of Igs appears to be non-selective, however, reduction of their absorption occurs in a gradually reducing rate from birth within the first 24 h after birth [10]. Rapid functional development of the small intestine is therefore essential and is guaranteed by a high rate of cell migration within the mucosal layer and expansion of intestinal mass (by approximately 40–60%). Furthermore, protein synthesis and enzymatic activity increase in response to colostrum intake [10].

#### 3.3.1. Immunoglobulins

After colostrum intake, there is transportation of lgs, growth factors and hormones into the lymphatics and general circulation [6,61,62,63,64]. Absorption of intact colostrum components through transcytosis is facilitated by enterocytes and specialized epithelial cells (microvillus or microfold-M cells) in the enteric epithelium that are known to develop during early embryogenesis [64,65]. Microvillus cells form part of the follicle-associated epithelium that surrounds Peyer’s patches and lymphoid follicles along the small intestine and appear to differentiate under stimulation of B lymphocyte cells.

Furthermore, these cells possess interspaced microvilli in their apical membrane that engulf macromolecules and antigens as part of their role in mucosal defence, while the basolateral membrane inverts forming pockets that harbour lymphocytes [64,65,66]. The first layer of epithelial intestinal tissue is comprised mainly of absorptive enterocytes, M cells and goblets cells followed by a layer of lymphoid tissue (Peyer’s patches, lymphoid follicles), another layer of dendritic cells and underlying adipocytes. Adipocytes have paracrine interaction with lymphocytes in order to provide them with fatty acids as an energy source for functioning. Dendritic cells promote lipolysis, which under local immune stimulation will modify the composition of the mobilized fatty acids [67].

Enzyme (protease) inhibitors present in colostrum, and selective adherence of colostrum immunoglobulins to M cells contribute to an effective transfer while the initial enhanced permeability of the intestinal epithelium declines. As the enterocyte matures increased intraluminal proteolysis gradually occurs between 36 and 48 h after birth [63].

#### 3.3.2. Cytokines

Cytokines are immunologic hormones that play an important role in the development of the foetal immune response and are also contained in colostrum. Cytokines present in bovine colostrum (interleukins, tumour necrosis factor β -TNFβ- and interferon γ) have been proposed to aid in lymphocyte recruitment during immune development after birth, as promoters of M cell differentiation reinforcing mucosal immunological defence [56,66]. The phagocytic capability of neutrophils has also shown to be improved after colostrum ingestion allowing for a more effective antibacterial protection [56].

#### 3.3.3. Immune Cells

Cells transferred by colostrum represented mainly by leukocytes (macrophages, lymphocytes and neutrophils) reach highest concentrations at about 24 h after birth. Maternal leukocytes will facilitate the development of antigen-presenting cells that represent the acquired immune response to antigens (infectious or vaccine inoculated) [54,60]. The antigen-specific responses measured in a calf following ingestion of maternal colostrum are related to specific immune memory in the dam [68]. Furthermore, in pigs [69], cell-mediated immunity produced by leukocytes is greatest when the colostrum contains abundant leukocytes. It is important to consider, however, that any treatment aiming at reducing the bacterial load of colostrum (e.g., heating or freezing) affects colostrum leukocytes decreasing their availability for the neonate [70].

#### 3.3.4. Growth Factors

Insulin-like growth factors (IGFs) are involved in immune response and growth regulation by enhancing early neonatal immune responses. Furthermore, IGFs are also implicated in inflammatory processes [55,56]. Absorption of colostrum IGFs and GH is facilitated by the low gastric acidity and almost no proteolytic digestion activity during the first hours after birth that allows growth factors to reach the intestine undigested. There, they are absorbed similarly to the immunoglobulins to enter neonatal circulation [63]. IGF type I and II present in colostrum are efficiently absorbed at low acidity at the apical membrane of the enterocyte and exert their action on cellular differentiation and growth facilitated by immune factors present in colostrum that safeguard the integrity of the mucosa [10,71]. Calves fed colostrum alone versus transitional milk or bulk tank milk showed better Ig and growth factor absorption and increased intestinal crypt and villi development [71].

Growth interacts in a compensatory fashion with other physiological processes such as immunity and reproduction in order to maintain survival of the individual [72]. Various studies investigating the relationship between the immune system and growth in concluded that the cost of maintaining an immune response is proportional to lower growth [72]. Moreover, maintenance of the immunity in the gut during early life has been found to have priority over body growth especially when animals are subjected to suboptimal diets and heavily contaminated environments [72].

#### 3.3.5. Somatotropic Axis Activation and Post-Natal Body Growth

Neonatal growth is influenced by three main factors: genetic background, nutritional intake and the environment. However, the endocrine system regulates animal growth and key processes of nutrient intake and metabolism [73,74]. In cattle, GH is released from the hypophysis under the influence of neurotransmitters, hypothalamic peptides and circulating metabolites. Although a sexual dimorphism in GH secretion patterns exists with a discrete pulse of GH with low inter pulse levels in males, and a high interpeak levels with less a pulsatile pattern in female [17], growth patterns and IGF-I concentration levels in serum have been related to GH peak amplitude and not to levels between pulses [75]. Optimal function of these axes is dependent on adequate dam and neonate nutrition.

In normal calves, GH release is controlled by GH releasing hormone (GHRH), as a stimulator, and somatostatin produced by the hypothalamus, pancreas and gastrointestinal tract as a release-inhibitor (Somatostatin release inhibitor factor-SRIF) [76]. An additional regulatory peptide (GH secretagogue), discovered during the 90’s, is ghrelin [74,77], for which its secretion is induced by fasting and suppressed by feeding [76]. This is a growth-hormone-releasing peptide (GHRP) that is produced in the hypothalamus, pituitary gland and the mucosal endocrine cells of the stomach. Therefore, it represents an endocrine link between the stomach, hypothalamus and pituitary gland regulating energy expenditure, appetite and the GH axis by increasing pulsatile secretion of GH in conjunction with growth-hormone-releasing hormone (GHRH). Hence, metabolic factors are implicated as GH secretion regulators, consistently with the role of GH in regulating metabolism and growth [75]. A direct correlation between colostrum GH and serum GH after colostrum intake has not been found but a regulatory effect of circulating IGF-I on animal growth has been demonstrated by Kerr et al., evidencing GH regulatory action on IGF-I production [77]. However, as reported in the review by Ontsouka et al. [78], mRNA transcripts encoding receptors of GH (GHR) have been identified in the gastrointestinal tissues of new-born calves; thus, implying that the physiological effects of GH, such as regulation of organ growth and development, and modulation of the calf ’s energy balance, can be mediated through intestinal GHR and, therefore, by the colostrum GH.

At puberty, leptin levels correlate to body fat reserves and ghrelin regulates feed intake [79]; two channels indicating body energy status, while growth is on a plateau state allowing for the unlocking of additional physiological processes such as the reproductive function.

#### 3.3.6. Factors Affecting Attainment of Puberty

Puberty is directly related to growth and comprises a gradual process to attaining reproductive competence. It involves intricate pathways that integrate internal endocrine and metabolic processes and external signals to activate the hypothalamic-pituitary-gonadal (HPG) axis [80]. Thus, reproductive function is initiated when there are adequate body fat reserves. Increased body weight gain advances puberty in heifers through the reduction of NPY expression on the arcuate nucleus of the hypothalamus and its projections onto GnRH and kisspeptin cells unlocking its inhibitory effects [81,82]. The effect of ghrelin on the hypothalamus regarding the attainment of puberty is not yet fully understood but it is thought that it mediates the suppressive effect of negative energy balance at the initiation of puberty [83].

Contributing molecules that have permissive action through an energy-signalling role at hypothalamic level are glucose, fatty acids, insulin, insulin-like growth factor I, thyroid hormones and growth hormone and act in connection with leptin and ghrelin [79,80,84,85].

There are recent published studies that report on the effects, at growth and reproductive development level, of administering an increased nutritional plane during the pre-weaning and postweaning period. Exciting results were shown in relation to metabolic indicators, during the postweaning phase with an increase in insulin, glucose and IGF-I as well as on dry matter intake and metabolizable energy [86]. In a second study, the same group reported improvements on reproductive tract development and function as carry-over effects due to increases on the plane of nutrition during the same periods [87].

Specifically, during the pre-weaning phase increases from 5 to 10 L/d of whole milk would rise leptin levels and LH pulses (amplitude, peak and duration) and an increased nutritional plane (from 70 to 85% concentrate) during the postweaning period would increase leptin levels. Moreover, a relation was established in which for every 1 ng/mL increase of leptin there could be a reduction of 22 days to attainment of puberty [88].

## 4. Strategies to Improve Immune Response, Growth and Attainment of Puberty in Calves

Many researchers do not focus on dam nutrition or colostrum feeding strategies for improving cattle productivity. Only recently has foetal programming, and the role of nutrient manipulation during late gestation, garnered scientific interest because of potential benefits for the offspring. For instance, research shows that low protein diets during late gestation hinder foetal growth, compromise glucose regulation in early life, and affect IGF-I serum concentration of new-born calves [89]. Whereas, protein supplementation increases birth weight, and hastens attainment of puberty when provided in the last trimester [40]. Furthermore, supplementing pregnant cows with rumen-protected methionine in late-pregnancy alters neonatal gene expression for gluconeogenesis, insulin signalling pathway, fatty acid oxidation [90,91], all of which improve survival. Another strategy, proposed by Garcia et al. [11], is to supplement unsaturated fatty acids during late gestation to achieve higher dry matter intake and average daily gain in the offspring. The higher weight gain was accompanied by increased glucose, IGF-I and lymphocytes in circulation. These animals also produced higher levels of interferon γ at 30 days of life, which is thought to be a reliable indicator of early immune system maturation [11]. In addition, feeding conjugated linoleic acid (CLA) to periparturient cows reduces negative energy balance, non-esterified fatty acids (NEFA), β-hydroxybutyrate (BHB) levels in blood and increases plasma levels of IGF-I [92]. This promotes growth in the offspring by activating the somatotropic axis, inducing gastrointestinal tract development and improving feed efficiency [11,93]. After birth, different recommendations have been proposed for colostrum supplementation. As reported by Godden et al. [70] factors influencing successful colostrum management include minimizing bacterial contamination of colostrum, the use of colostrum supplementation and replacers, benefits of multiple feedings, and benefits of extended colostrum or transition milk feeding after intestinal closure [57]. Among those strategies, dairy calves be fed 3 L of good-quality colostrum [94,95] (classified as containing at least >50 g/L of IgG [96]) within 2 h of birth by oesophageal tube or at least 2 L within 4 h and a total of 4 L within 12 h from birth by nipple feeding. Because this approach doesn’t take into account body weight variations, it has been suggested to feed colostrum at 8.5% of the calves’ body weight (BW) within 2 h of birth in order to achieve a greater concentration of IgG in serum in the first 3 days of life than feeding either 7 or 10% of BW in order to improve overall calf health and growth [97]. Furthermore, the addition of supplemental colostrum to milk replacer for the first two weeks of life [98], or extending colostrum feeding, as inclusion into whole milk (700 g of colostrum in 5 kg of pasteurized whole milk) for 14 days after birth has been proved to improve the overall calves’ health and growth performance [99]. In addition, Pyo et al. [100] demonstrated that feeding either colostrum or a mixture 1:1 of colostrum and whole milk for 3 days promotes small intestinal maturation. Therefore, these studies suggest that feeding calves a mixture of colostrum and milk for a longer period may be a strategy to improve calf performance post-weaning.

## 5. Conclusions

Working towards eliminating health issues in young calves is a critical step of a One Health approach to ensuring food security. For profitable cattle production, there is a need to adopt strategies that ensure optimal performance of replacement heifers. Taking advantage of pregnancy as the first available and, possibly, most influential developmental window, by providing and/or reinforcing adequate nutritional and energy requirements, will positively impact future offspring performance by unlocking full genetic potential. This will not only enhance ante-natal interactions but will enrich colostrum which will in turn promote immune system functionality, intestinal development and better growth rate on the neonate.
